# Determination of band offsets, hybridization, and exciton binding in 2D semiconductor heterostructures

**DOI:** 10.1126/sciadv.1601832

**Published:** 2017-02-08

**Authors:** Neil R. Wilson, Paul V. Nguyen, Kyle Seyler, Pasqual Rivera, Alexander J. Marsden, Zachary P.L. Laker, Gabriel C. Constantinescu, Viktor Kandyba, Alexei Barinov, Nicholas D.M. Hine, Xiaodong Xu, David H. Cobden

**Affiliations:** 1 Department of Physics, University of Warwick, Coventry CV4 7AL, U.K.; 2 Department of Physics, University of Washington, Seattle, WA 98195, USA.; 3 Theory of Condensed Matter Group, Cavendish Laboratory, University of Cambridge, 19 JJ Thomson Avenue, Cambridge CB3 0HE, U.K.; 4 Elettra-Sincrotrone Trieste S.C.p.A., Basovizza, 34149 Trieste, Italy.; 5 Physics Department, University of Trieste, Via Valerio 2, 34127 Trieste, Italy.; 6 Department of Materials Science and Engineering, University of Washington, Seattle, WA 98195, USA.

**Keywords:** μ-ARPES, photoluminescence, linear-scaling DFT, 2D semiconductor, Graphene, heterobilayer, electronic properties, band hybridization, commensuration

## Abstract

Combining monolayers of different two-dimensional semiconductors into heterostructures creates new phenomena and device possibilities. Understanding and exploiting these phenomena hinge on knowing the electronic structure and the properties of interlayer excitations. We determine the key unknown parameters in MoSe_2_/WSe_2_ heterobilayers by using rational device design and submicrometer angle-resolved photoemission spectroscopy (μ-ARPES) in combination with photoluminescence. We find that the bands in the K-point valleys are weakly hybridized, with a valence band offset of 300 meV, implying type II band alignment. We deduce that the binding energy of interlayer excitons is more than 200 meV, an order of magnitude higher than that in analogous GaAs structures. Hybridization strongly modifies the bands at Γ, but the valence band edge remains at the K points. We also find that the spectrum of a rotationally aligned heterobilayer reflects a mixture of commensurate and incommensurate domains. These results directly answer many outstanding questions about the electronic nature of MoSe_2_/WSe_2_ heterobilayers and demonstrate a practical approach for high spectral resolution in ARPES of device-scale structures.

## Teaser

Photoemission measurements on exfoliated 2D heterostructures reveal detailed electronic structure and hybridization effects.

## INTRODUCTION

A variety of van der Waals heterostructures have recently attracted attention, including graphene/hBN (hexagonal boron nitride) for its unusual electronic structure (*1*), graphene/TMD (transition metal dichalcogenide) (*2*) and TMD/TMD for efficient photocurrent generation (*3*–*5*), and graphene/hBN/TMD for light-emitting diodes (*6*). In semiconducting TMD heterobilayers, ultrafast charge transfer (*7*) and formation of interlayer excitons with the electron and hole in opposite layers (*8*) have been observed. Such heterobilayers are also predicted to host rich valley physics (*9*), and, promisingly, valley polarization of the interlayer excitons has been seen (*10*) in aligned (small twist angle) heterobilayers of WSe_2_ and MoSe_2_, two isostructural semiconductors that are closely lattice-matched (*11*).

Although optical and transport studies have made rapid progress, there are still many open questions that cannot be addressed by these techniques alone, including the following: Does a semiconductor heterobilayer have a direct bandgap at the K points? To what extent do the orbitals hybridize? Can one regard the bands at K as simply being those from isolated monolayers? What are the band offsets that govern the nature and binding energy of interlayer excitons? Are the layers in the heterobilayer commensurate? Angle-resolved photoemission spectroscopy (ARPES) has the potential to answer these questions. It has been used extensively to determine two-dimensional (2D) band structures in large-area van der Waals structures (*12*–*18*). However, 2D semiconductor heterostructures are currently limited to a few micrometers in size, necessitating the use of μ-ARPES techniques (*19*, *20*). Here, by introducing a sample design that affords an order of magnitude higher spectral resolution in μ-ARPES (<50 meV) than in previous studies, we have been able to answer all of the above questions for the canonical MoSe_2_/WSe_2_ system.

## RESULTS

### Approach and sample design

**Fig. 1:**
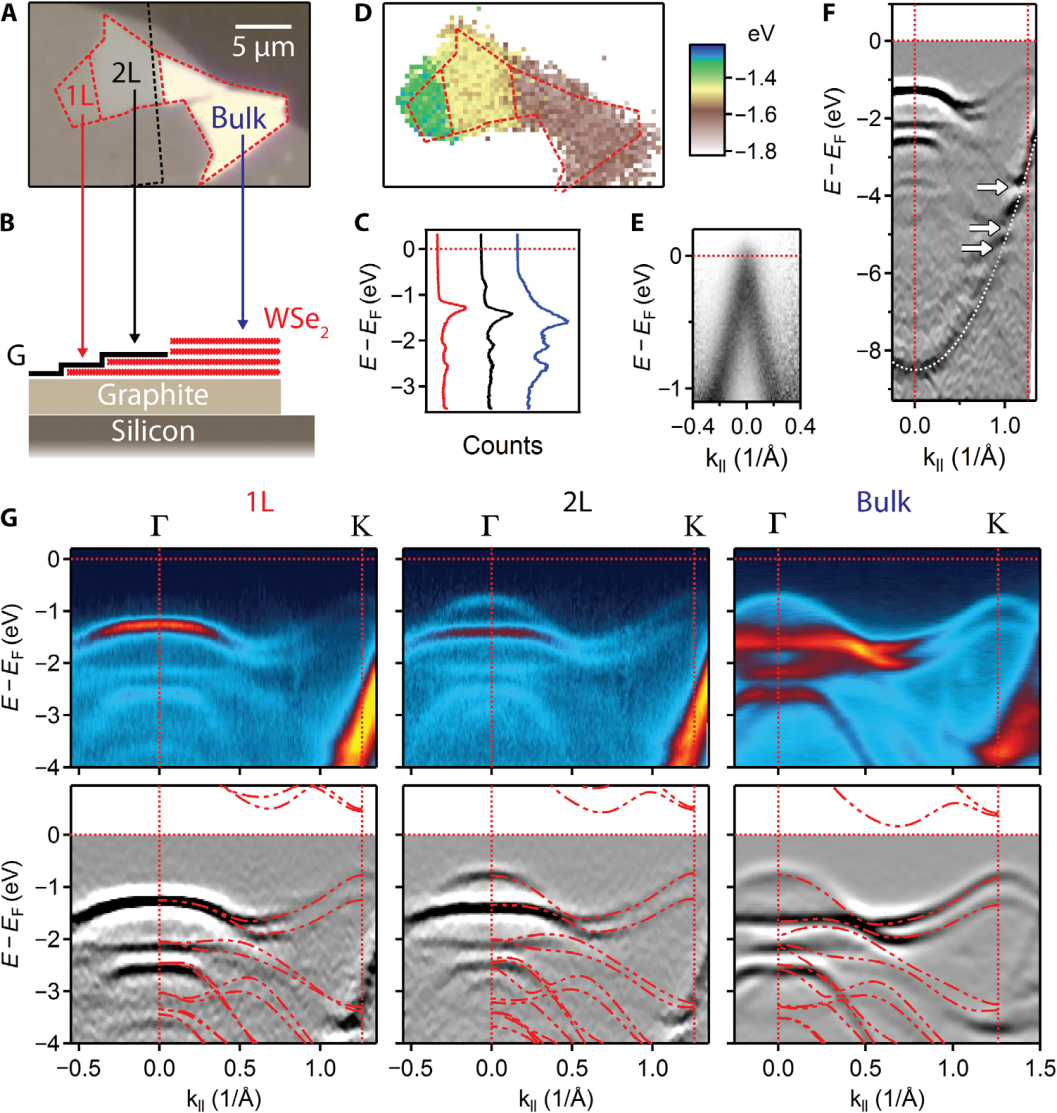
Bands and hybridization in graphene-encapsulated WSe_2_ measured by μ-ARPES. (**A**) Optical image and (**B**) schematic cross section of an exfoliated WSe_2_ flake with monolayer (1L), bilayer (2L), and bulk regions partially capped with monolayer graphene (G) and supported by a graphite flake on a doped silicon substrate. (**C**) Angle-integrated spectra from each region in (A). (**D**) Map of the energy of peak emission, showing contrast between 1L, 2L, and bulk regions. (**E**) Momentum slice through the graphene K point, showing that *E*_F_ is at the Dirac point. (**F**) Momentum slice along Γ − K (WSe_2_) in the 1L region. The intensity is twice-differentiated with respect to energy. Avoided crossings between the graphene valence band (white dotted line) and the monolayer WSe_2_ bands are indicated by white arrows. (**G**) Momentum slice of unprocessed (top) and twice-differentiated ARPES (bottom) along Γ − K (WSe_2_) in the 1L (left), 2L (middle), and bulk (right) regions. Below is the intensity twice-differentiated with respect to energy with overlaid DFT calculation (red dashed lines).

To illustrate our approach and demonstrate its effectiveness, we first studied the effect of hybridization between monolayers of WSe_2_. The optical image (Fig. 1A) shows an exfoliated WSe_2_ flake that naturally has monolayer (1L), bilayer (2L), and multilayer (bulk) regions; their boundaries are indicated by red dashed lines. Figure 1B is a schematic cross section. The flake is partly capped by a graphene monolayer (G), outlined by a black dashed line, which is essential for the sample to be annealed at 400°C in high vacuum to remove surface contamination without degrading the TMD beneath it. It rests on a thin graphite flake exfoliated directly onto a p-doped silicon chip that serves as an atomically flat conducting substrate (fig. S1). Contamination that is trapped between the layers during transfer collects in blisters, which consolidate upon annealing, leaving the remainder of the interfaces atomically clean (*21*). The sample is located by scanning photoemission microscopy (SPEM) using an approximately 1-μm beam spot at 74 eV photon energy (see Materials and Methods).

Figure 1C shows momentum-integrated spectra taken at points in each region of the WSe_2_ flake. The highest intensity peak shifts downward monotonically in energy as the number of layers increases. A SPEM map of the peak energy versus location (Fig. 1D) therefore shows contrast between the 1L, 2L, and bulk regions. All spectra were highly consistent within each region, with no spatial variations that would signal fixed charges from contamination or in the substrate, and no drift due to charging resulting from photoemission was detected. From momentum-resolved energy slices, we could determine the orientations of the WSe_2_ flake, graphene cap, and graphite support (fig. S2). Figure 1E shows a momentum slice through the graphene K point in the 1L region. The Dirac point energy *E*_D_ coincides with the Fermi level *E*_F_ (red dotted line) to within the measurement accuracy of <50 meV, implying minimal charge transfer between WSe_2_ and graphene or doping of other origin. This, in turn, implies that there is no significant density of defect states in the gap of the WSe_2_. Figure 1F shows the second derivative of a momentum slice along Γ-K(WSe_2_) in the 1L region. The valence band of the capping graphene is marked by a white dotted curve. It hybridizes with the WSe_2_ bands, producing avoided crossings (white arrows) similar to those seen in graphene on MoS_2_ (*18*). These features are >3 eV below *E*_F_, and the important WSe_2_ bands nearer *E*_F_ (*22*) are not affected.

Figure 1G presents Γ-K slices showing the important features within 4 eV of the Fermi level for the 1L, 2L, and bulk WSe_2_ regions, along with their second derivatives. All features of the upper bands are well resolved. The spectra are consistent with expectations based on the literature (*23*), and density functional theory (DFT, overlaid red dashed lines) reproduces the upper valence band well, with no adjustable parameters other than an energy offset chosen to match the uppermost measured band at Γ. The bands near K are almost unchanged from monolayer to bulk (*22*, *24*) because of their in-plane orbital character (W 5*d*_*xy*_ and 5$d_{x^{2}-y^{2}}$), and in the monolayer (*23*, *25*), the valence band edge is at K. On the other hand, there are strong hybridization effects on the bands near Γ because of their out-of-plane orbital character (Se 4*p*_*z*_ and W 5$d_{z^{2}}$). In the bilayer and the bulk, the valence band splits at Γ with a higher-mass band 0.25 eV below that in the monolayer and a lower-mass band that is 0.50 eV higher. In the bilayer, the valence band edge is still at K, whereas in the bulk, it moves to Γ.

### MoSe_2_/WSe_2_ heterostructures

**Fig. 2:**
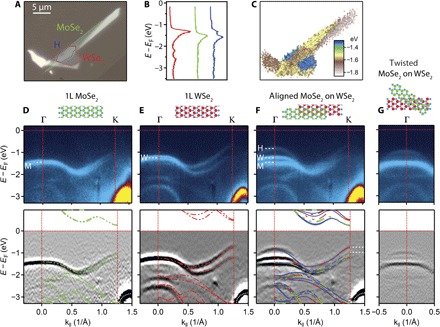
Bands in a 2D heterostructure. (**A**) Optical image showing monolayer MoSe_2_ and WSe_2_ sheets, which overlap, with the MoSe_2_ on top, in an aligned heterobilayer region (H). Their boundaries are indicated with color-coded dotted lines. (**B**) Angle-integrated spectra in each of the three regions. (**C**) Map of the energy of maximum emission. (**D** to **F**) Momentum slices along Γ − K in the three regions, (top) unprocessed and (bottom) twice-differentiated, with cartoons of the structures above. The superposed dashed colored lines are DFT calculations for the MoSe_2_ monolayer (green), the WSe_2_ monolayer (red), and the commensurate heterobilayer (blue). The graphene valence band is indicated by a white dotted line. The white dashes in the lower panel of (F) indicate the valence band maxima in the MoSe_2_ and WSe_2_ monolayers and hence the valence band offset. The white dashed lines in the upper panels of (D) to (F) mark the valence band maxima in the isolated MoSe_2_ (M) and WSe_2_ (W) monolayers and in the aligned heterobilayer (H). (**G**) A momentum slice near Γ in another heterobilayer intentionally misaligned by about 30°. Here, only two bands are seen, indicating that the third band near Γ in the aligned heterobilayer (F) arises from commensurate domains.

We now turn to the central object of our study, semiconductor heterobilayers. Figure 2A is an optical image of a sample with a MoSe_2_ monolayer (green dashed line) partially overlapping a WSe_2_ monolayer (red dashed line), forming a heterobilayer region (H) (blue dashed line). The monolayers were aligned during transfer by identifying the crystal axes using polarization-resolved second-harmonic generation (fig. S3) (*26*–*28*). As before, we included a protecting graphene cap and a graphite support. Figure 2B shows angle-integrated photoemission spectra from one point in each region. The largest peak is ~200 meV lower in the MoSe_2_ monolayer than in the WSe_2_ monolayer, whereas in the H region, there are two peaks that are shifted relative to the monolayer peaks. As a result, a map of the energy where the intensity is highest versus position (Fig. 2C) shows contrast between monolayer and H regions. In constant-energy slices, the K points of the two monolayers coincide in momentum space (fig. S4), confirming a twist angle of less than 1° and consistent with lattice constants differing by <1%.

The variation in band structure across the heterojunction is seen in the Γ-K momentum slices in Fig. 2 (D to F) for 1L MoSe_2_, 1L WSe_2_, and the heterobilayer, respectively. The upper valence bands in the monolayer regions are again well matched by DFT (green and red dashed lines). The spin-orbit splitting at K is much smaller in the MoSe_2_ than in the WSe_2_, and the valence band edge is substantially lower. In the heterobilayer, the bands near K are very similar to the bands in the monolayers, implying weak interlayer hybridization near K, as was the case for the WSe_2_ homobilayer. On the other hand, the bands at Γ are substantially different from those in the monolayers, implying significant hybridization, again as in the WSe_2_ homobilayer. Nevertheless, the valence band edge remains at K. This is important for the electrical and optical properties.

Interestingly, we clearly see three bands within 0.5 Å^−1^ of Γ, not just the two that would be expected from homogeneous hybridization of one band from each monolayer. We note, however, that the third band resembles the upper band in the WSe_2_ homobilayer (Fig. 1G), in which the layers are perfectly commensurate, having the bulk 2H stacking. We also recall that when monolayers with mismatched lattice constants are stacked, elastic energy considerations will ensure that any commensurate domains have a finite size. This has been demonstrated for graphene on hBN (*29*). For zero twist angle, the scale of the domains is $\frac{a^{2}}{\delta a}$, where *a* is the lattice constant and δ*a* is the difference. Here, this scale is ~100 nm, which is less than the x-ray spot size. The spectrum of the heterobilayer could thus be interpreted as a superposition of spectra from a mixture of incommensurate domains in which hybridization is weak and commensurate domains in which hybridization is similar to that in the homobilayer.

In support of this interpretation, DFT simulations of the commensurate heterobilayer reproduce the uppermost band at Γ (blue lines) (Fig. 2F) and the slightly downward shifted lower band. Adding the hybridized bands of the isolated MoSe_2_ and WSe_2_ monolayers (green and red lines, respectively) reproduces the three apparent bands in H fairly closely. The remaining small discrepancy can be accounted for by shifts on the order of 100 meV in the incommensurate case, roughly independent of twist angle (*30*), as predicted by linear-scaling DFT (fig. S5) (*31*). Additionally, in an intentionally misaligned (by ~30°) MoSe_2_/WSe_2_ heterobilayer, where no commensuration is expected, we saw only two bands near Γ, as illustrated in Fig. 2G and fig. S6. The band shifts in the twisted heterobilayer are well matched by DFT predictions for incommensurate layers (fig. S7). Furthermore, in a sample with an aligned bilayer of MoSe_2_ on a monolayer of WSe_2_, we observed four bands at Γ rather than three (fig. S8). The combined evidence that aligned heterobilayers are composed of mixtures of incommensurate and commensurate domains is therefore compelling.

**Fig. 3:**
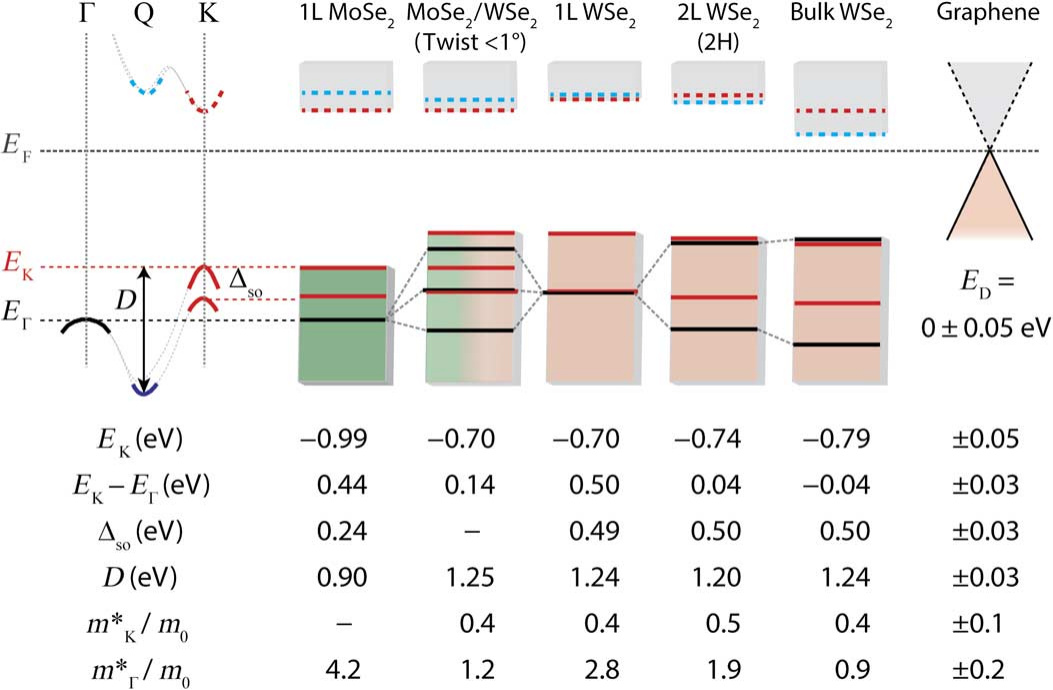
Summary of measured band parameters. Left: Schematic showing the definitions of parameters applicable for monolayers and aligned bilayers. Solid lines signify measured quantities, and dotted lines denote DFT calculations. Main: Graphical illustration of the positions of homologous band edges and hybridization effects. In both 2L WSe_2_ and heterobilayer MoSe_2_/WSe_2_, hybridization is almost undetectable at K (red) but much larger at Γ (black). Bottom: Table of quantities determined by fitting the μ-ARPES spectra shown in Figs. 1 and 2. Energies are from Lorentzian fits to the second-derivative curves. The effective masses, which are isotropic within the accuracy of the fits, are obtained from weighted parabolic fits to the above band positions in symmetric windows about K and Γ with widths of 0.08 Å^− 1^ and 0.15 Å^− 1^, respectively.

The values of key parameters extracted from the μ-ARPES measurements are summarized in Fig. 3. They were consistent across multiple samples and showed no dependence on the orientation of the graphene cap or graphite substrate. The spin-orbit splitting Δ_SO_ at K is 0.49 ± 0.03 eV in WSe_2_ and 0.24 ± 0.03 eV in MoSe_2_, in agreement with the literature (*23*), as are the effective masses of holes at Γ and K. In the WSe_2_ monolayer, we find *E*_K_ − *E*_Γ_ = 0.50 ± 0.03 eV, consistent with scanning tunneling spectroscopy results (*32*), and in the MoSe_2_ monolayer, we find *E*_K_ − *E*_Γ_ = 0.44 ± 0.03 eV. We also record here the valence band width *D*, which is useful for comparison with band structure calculations (*23*). As is well known, in both monolayer species, the valence band edge is at K, whereas in the bulk, it is at Γ. In the heterobilayer, we find that the valence band edge is also at K and is higher than the maximum at Γ by 0.14 ± 0.03 eV. We measured a valence band offset (VBO) between the WSe_2_ and MoSe_2_ monolayers of Δ_VBO_ = 0.30 ± 0.03 eV. Because the bands at Γ in H (Fig. 2F) align well with those in the separate monolayers, we infer that this value is an intrinsic parameter of the heterojunction and that any charge transfer between the layers has negligible effect on the measurement.

Because we cannot probe the conduction band and the single-particle gaps have not been established incontrovertibly, we show the conduction band edges at K (red dashed line) and Q (blue dashed line) calculated using DFT. Although DFT underestimates these energies, the predictions of variations within the family of materials and across the Brillouin zone are more reliable (*23*, *24*). The conduction band edge in H is predicted to remain at the K point, which, together with our measurements, implies that the band gap in H is direct.

### Interlayer exciton binding energy

**Fig. 4:**
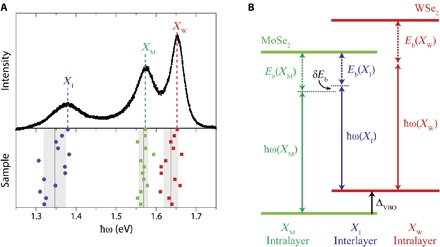
Photoluminescence and exciton binding in aligned MoSe_2_/WSe_2_ heterobilayers. (**A**) Top: Representative photoluminescence spectrum showing peaks due to intralayer (*X*_M_ and *X*_W_) and interlayer (*X*_I_) excitons (excitation of 2.33 eV at 20 μW). Bottom: Peak positions for 13 samples, implying that the energy of *X*_I_ is 220 ± 20 meV below that of *X*_M_. (**B**) Energy diagram showing the connection between the three exciton energies and the levels derived from the MoSe_2_ and WSe_2_ conduction and valence bands at the K points.

We can gain important insights into exciton binding by combining these results with optical measurements. Figure 4A shows a photoluminescence spectrum from an aligned WSe_2_/MoSe_2_ heterobilayer sample at room temperature. Below it is a plot of the peak positions for 13 similar samples. There are three peaks, whose origins are indicated schematically in Fig. 4B. *X*_M_ and *X*_W_ are the intralayer excitons formed by an electron and a hole in bands from the same layer, either MoSe_2_ or WSe_2_, respectively. Their energies ℏω(*X*_M_) and ℏω(*X*_W_) are almost coincident with the corresponding valley excitons in the isolated monolayers, consistent with the observation that the band-edge states near the K points barely hybridize and implying that the binding energy of intralayer excitons in one layer is insensitive to the presence of the other layer. The third peak is the interlayer exciton *X*_I_. The small (~2%) variation of ℏω(*X*_I_) between samples could be due to variations in substrate doping or twist angle.

According to Fig. 4B, the energy difference between the intralayer and interlayer excitons has two contributions: the difference in their binding energies δ*E*_b_ = *E*_b_(*X*_M_) − *E*_b_(*X*_I_) and the valence band offset, such that ℏω(*X*_M_) − ℏω(*X*_I_) = Δ_VBO_ − δ*E*_b_. The uniformity of *ℏ*ω(*X*_I_) is consistent with Δ_VBO_ being an invariant parameter of the heterojunction. Hence, by combining optical and ARPES measurements made at the same temperature, we can deduce the magnitude of δ*E*_b_ = ℏω(*X*_I_) − ℏω(*X*_M_) + Δ_VBO_. Averaging over the samples, we get ℏω(*X*_M_) − ℏω(*X*_I_) = 0.22 ± 0.02 eV, at 300 K. At 105 K, it is slightly larger, by about 0.03 eV (see section S7). Then, using Δ_VBO_ = 0.30 ± 0.03 eV from above gives δ*E*_b_ = 0.05 ± 0.04 eV. That *X*_I_ is more weakly bound than *X*_M_ is not surprising because the electron and hole in different layers are, on average, further apart. The reported values of *E*_b_ for similar monolayers range from ~0.3 to 0.7 eV (*33*–*40*), with a value of 0.55 eV for MoSe_2_ (*33*). We deduce that the interlayer binding energy *E*_b_(*X*_I_) = *E*_b_(*X*_M_) − δ*E*_b_ is at least ~0.2 eV. This is an order of magnitude larger than the binding energy of spatially indirect excitons in GaAs/AlGaAs double quantum wells.

## DISCUSSION

The results described above establish the key electronic parameters of MoSe_2_/WSe_2_ heterobilayers. The hybridization effects at Γ provide the first evidence for commensurate domains in such heterostructures, suggesting the possibility of band engineering by layering similar to that discussed in the context of graphene on hBN. Confirmation of this explanation will, however, require further research, such as higher-resolution ARPES measurements showing the absence of hybridization of bands from the spatially separated domains. The observations that the valence band edge remains at the K point and that the band alignment is type II are both significant for electronic and optoelectronic applications.

Electron doping is required to probe the conduction band of insulators by ARPES. Our samples are undoped, but the sample design offers the possibility of gate doping in situ in the ARPES chamber. In cases where the bands of the graphene cap may obscure features near the Fermi energy, the graphene can be replaced with monolayer hBN, which is equally effective but harder to work with.

It is clear that the technique of μ-ARPES combined with careful sample design provides invaluable information for realizing the potential of 2D semiconductor heterostructures. It will enable the local electronic structure and chemical potential to be determined in all types of other 2D materials and devices.

## MATERIALS AND METHODS

Samples were fabricated by exfoliation and dry transfer, as detailed in figs. S1 and S2. μ-ARPES was performed at the Spectromicroscopy beamline of the Elettra light source, with linearly polarized radiation focused to an approximately 0.6-μm-diameter spot by a Schwarzschild objective (*41*) and incident at 45° with respect to the sample. The energy and momentum resolution of the hemispherical electron analyzer were ~50 meV and ~0.03 Å^−1^, respectively. SPEM maps were acquired over the energy range of the fixed detector (~3.5 eV at the pass energy used), integrating over its angular range of ~15° (at 70 eV, this is ~1.1 Å^−1^). Samples were annealed at up to 700 K for a total of 1 to 2 hours in ultrahigh vacuum before measurement to remove adsorbates that accumulated during exposure to the atmosphere. In some cases, repeating this anneal modestly improved the resolution. The sample temperature during measurements was 110 K.

Photoluminescence measurements were performed using ~30 μW of 532- or 632.8-nm continuous-wave laser excitation in reflection geometry, with the signal collected by a spectrometer and a Si charge-coupled device. Measurements described above were performed in an ambient environment, and additional low-temperature measurements (fig. S9) were completed in vacuum in a closed-cycle cryostat.

The Quantum Espresso plane-wave DFT package (*42*) was used for calculating individual materials and aligned heterostructures (Figs. 1 to 3), including the spin-orbit interaction (*43*). For simulations involving twisted heterostructures, the ONETEP linear-scaling DFT code (*31*) was used. Further details are given in sections S3 and S7.

## Supplementary Material

http://advances.sciencemag.org/cgi/content/full/3/2/e1601832/DC1

## SUPPLEMENTARY MATERIALS

Supplementary material for this article is available at http://advances.sciencemag.org/cgi/content/full/3/2/e1601832/DC1 section S1. Fabrication of encapsulated WSe_2_ and additional ARPES data section S2. Fabrication of and further ARPES from a MoSe_2_/WSe_2_ heterobilayer structure section S3. Linear-scaling DFT calculations for twisted MoSe_2_/WSe_2_ heterobilayers section S4. Band structure of twisted monolayer MoSe_2_/WSe_2_ section S5. ARPES of encapsulated MoSe_2_/WSe_2_ with heterotrilayer regions section S6. Exciton energies at lower temperatures section S7. DFT methodology fig. S1. Fabrication of a graphene, WSe_2_, and graphite heterostructure. fig. S2. Relative orientations of the graphene, WSe_2_, and graphite heterostructure. fig. S3. Fabrication of a MoSe_2_/WSe_2_ heterostructure. fig. S4. Relative orientations of the layers in an encapsulated MoSe_2_/WSe_2_ heterostructure. fig. S5. Linear-scaling DFT predictions of the band structure of the twisted MoSe_2_/WSe_2_ interface. fig. S6. Band structure of a twisted monolayer MoSe_2_/WSe_2_ heterostructure. fig. S7. Comparison between bands and hybridization in aligned and twisted heterostructures. fig. S8. Bands and hybridization in a MoSe_2_/WSe_2_ structure with heterotrilayer regions. fig. S9. Lower-temperature interlayer exciton photoluminescence. References (*44*–*50*)
