# Supplementary material for: Determination of band offsets, hybridization, and exciton binding in 2D semiconductor heterostructures
Source: Sci Adv. 2017 Feb 8;3(2):e1601832. doi: 10.1126/sciadv.1601832 (PMC5298850; doi:10.1126/sciadv.1601832)
Supplement: http://advances.sciencemag.org/cgi/content/full/3/2/e1601832/DC1 [file supp_3_2_e1601832__index.html]

Science Advances | Science Advances

## Supplementary Materials

**This PDF file includes:**

- section S1. Fabrication of encapsulated WSe2 and additional ARPES data
- section S2. Fabrication of and further ARPES from a MoSe2/WSe2 heterobilayer structure
- section S3. Linear-scaling DFT calculations for twisted MoSe2/WSe2 heterobilayers
- section S4. Band structure of twisted monolayer MoSe2/WSe2
- section S5. ARPES of encapsulated MoSe2/WSe2 with heterotrilayer regions
- section S6. Exciton energies at lower temperatures
- section S7. DFT methodology
- fig. S1. Fabrication of a graphene, WSe2, and graphite heterostructure.
- fig. S2. Relative orientations of the graphene, WSe2, and graphite heterostructure.
- fig. S3. Fabrication of a MoSe2/WSe2 heterostructure.
- fig. S4. Relative orientations of the layers in an encapsulated MoSe2/WSe2 heterostructure.
- fig. S5. Linear-scaling DFT predictions of the band structure of the twisted MoSe2/WSe2 interface.
- fig. S6. Band structure of a twisted monolayer MoSe2/WSe2 heterostructure.
- fig. S7. Comparison between bands and hybridization in aligned and twisted heterostructures.
- fig. S8. Bands and hybridization in a MoSe2/WSe2 structure with heterotrilayer regions.
- fig. S9. Lower-temperature interlayer exciton photoluminescence.
- References (*44–50*)

Download PDF

**Files in this Data Supplement:**

- Adobe PDF - 1601832\_SM.pdf
