# Supplementary material for: Determination of band offsets, hybridization, and exciton binding in 2D semiconductor heterostructures
Source: Sci Adv. 2017 Feb 8;3(2):e1601832. doi: 10.1126/sciadv.1601832 (PMC5298850; doi:10.1126/sciadv.1601832)
Supplement: http://advances.sciencemag.org/cgi/content/full/3/2/e1601832/DC1 [file 1601832_SM.pdf]

Science Advances

AAAS

advances.sciencemag.org/cgi/content/full/3/2/e1601832/DC1

Supplementary Materials for

Determination of band offsets, hybridization, and exciton binding in 2D semiconductor heterostructures

Neil R. Wilson, Paul V. Nguyen, Kyle Seyler, Pasqual Rivera, Alexander J. Marsden, Zachary P. L. Laker, Gabriel C. Constantinescu, Viktor Kandyba, Alexei Barinov, Nicholas D. M. Hine, Xiaodong Xu, David H. Cobden

Published 8 February 2017, *Sci. Adv.* **3**, e1601832 (2017)
DOI: 10.1126/sciadv.1601832

This PDF file includes:

- section S1. Fabrication of encapsulated WSe_2 and additional ARPES data
- section S2. Fabrication of and further ARPES from a $MoSe_2/WSe_2$ heterobilayer structure
- section S3. Linear-scaling DFT calculations for twisted $MoSe_2/WSe_2$ heterobilayers
- section S4. Band structure of twisted monolayer $MoSe_2/WSe_2$
- section S5. ARPES of encapsulated $MoSe_2/WSe_2$ with heterotrilayer regions
- section S6. Exciton energies at lower temperatures
- section S7. DFT methodology
- fig. S1. Fabrication of a graphene, WSe_2, and graphite heterostructure.
- fig. S2. Relative orientations of the graphene, WSe_2, and graphite heterostructure.
- fig. S3. Fabrication of a $MoSe_2/WSe_2$ heterostructure.
- fig. S4. Relative orientations of the layers in an encapsulated $MoSe_2/WSe_2$ heterostructure.
- fig. S5. Linear-scaling DFT predictions of the band structure of the twisted $MoSe_2/WSe_2$ interface.
- fig. S6. Band structure of a twisted monolayer $MoSe_2/WSe_2$ heterostructure.
- fig. S7. Comparison between bands and hybridization in aligned and twisted heterostructures.
- fig. S8. Bands and hybridization in a $MoSe_2/WSe_2$ structure with heterotrilayer regions.
- fig. S9. Lower-temperature interlayer exciton photoluminescence.
- References (*44–50*)

Supplementary Materials

section S1. Fabrication of encapsulated WSe_2 and additional ARPES data

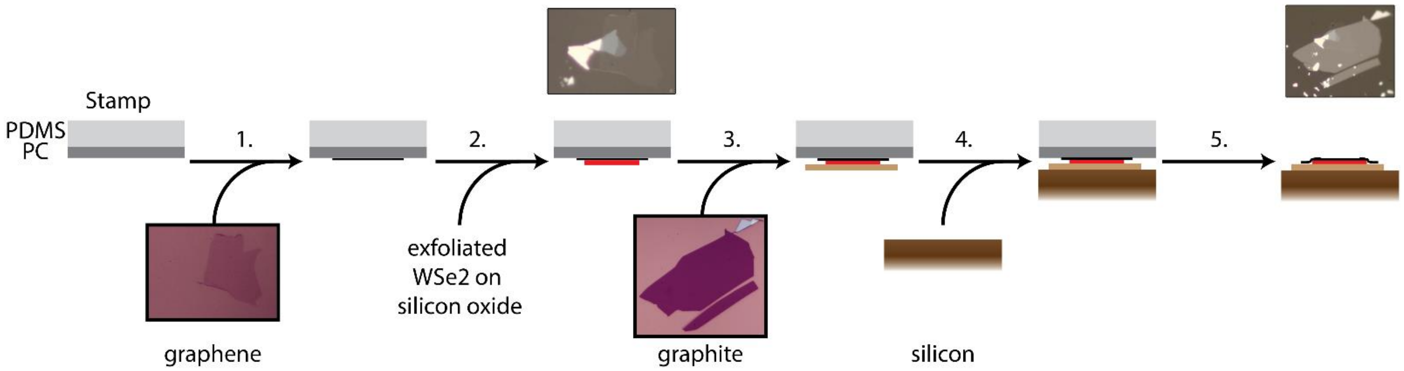

fig. S1. Fabrication of a graphene, WSe_2, and graphite heterostructure. Schematic of the sample fabrication process for the WSe_2 sample in Fig. 1 of the main text.

1. Graphene was exfoliated onto silicon oxide and a suitable flake identified by optical microscopy. The graphene flake was transferred onto a polycarbonate (PC) on polydimethylsiloxane (PDMS) stamp by dry transfer, (*44*) with a peak temperature of 105 °C to maximize graphene-to-PC adhesion. The stamp is on a glass slide mounted to a Märzhäuser Wetzlar (MW) SM 3.25 motorized micromanipulator controlled by joystick via an MW Tango controller.

2. WSe_2 was exfoliated onto silicon oxide and a suitable flake identified by optical microscopy. The graphene-on-stamp was aligned to the WSe_2 flake, heated to 90 °C against it, and then peeled off to remove the WSe_2 from the silicon oxide substrate, giving WSe_2-on-graphene-on-stamp.

3. A thin flake of graphite was exfoliated onto silicon oxide and identified by optical microscopy. The stamp was aligned to the graphite and dry transfer at 90 °C was again used to remove the graphite from the silicon oxide, giving a graphite-WSe_2-graphene stack on the stamp.

4. The stack and stamp were firmly placed on a doped silicon substrate.

5. The substrate was heated to 150 °C and the PDMS stamp peeled off, leaving the stack and PC on the silicon. The sample was then cleaned, removing the PC, by a solvent wash in chloroform, rinsed in IPA, and dried under N_2 gas followed by thermal annealing in an Argon (95%)/Hydrogen(5%) atmosphere at 400 °C for 2 hr.

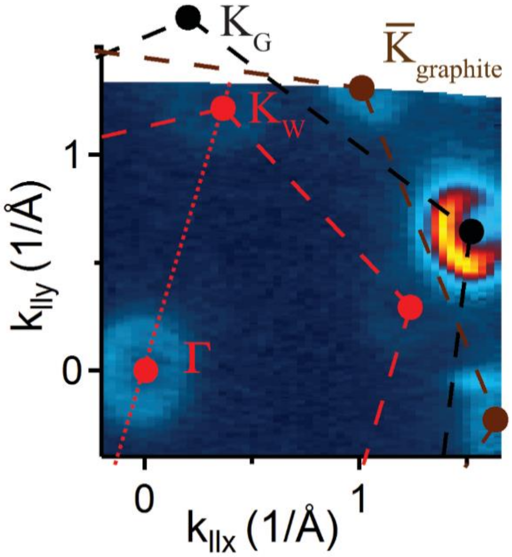

fig. S2. Relative orientations of the graphene, WSe_2, and graphite heterostructure. Measured by µ-ARPES. Constant energy slice at $\boldsymbol{E - E_F = 0.9}$ eV from the 1L WSe_2 region. The positions of the graphene (black), WSe_2 (red), and graphite (brown) K-points are labelled.

section S2. Fabrication of and further ARPES from a $MoSe_2/WSe_2$ heterobilayer structure

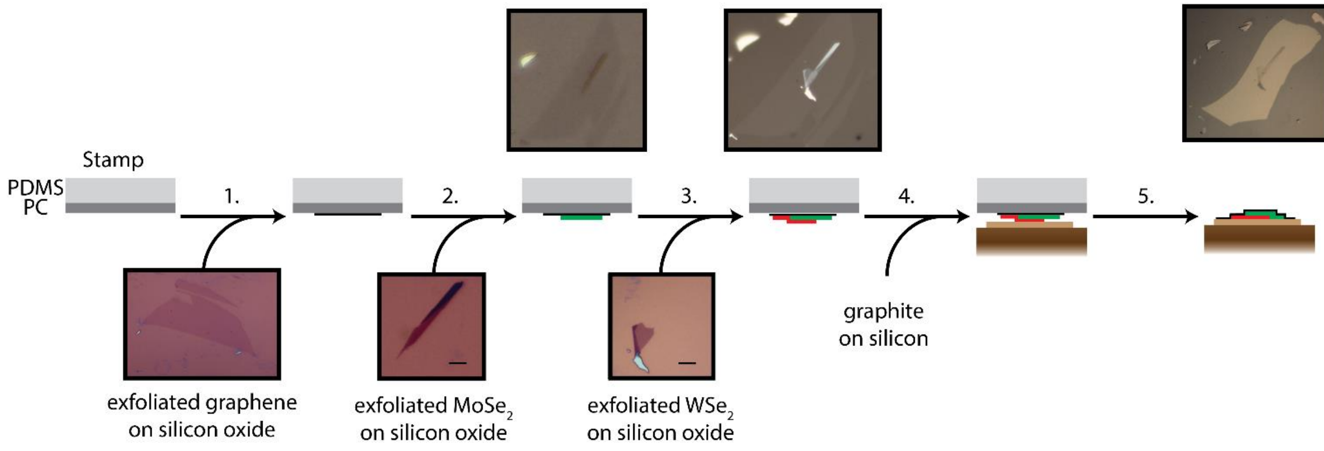

fig. S3. Fabrication of a $MoSe_2/WSe_2$ heterostructure. Schematic of the sample fabrication process for the $MoSe_2$-on-WSe_2 sample in Fig. 2 of the main text. Scale bars are 5 µm.

1. Graphene was exfoliated onto silicon oxide and a suitable flake identified by optical microscopy. The graphene flake was transferred onto a polycarbonate (PC) on polydimethylsiloxane (PDMS) stamp by dry transfer, (*44*) with a peak temperature of 105 °C to maximize graphene-to-stamp adhesion. The stamp is on a glass slide which is taped to a rod mounted to a Märzhäuser Wetzlar (MW) SM 3.25 motorized micromanipulator controlled by joystick via an MW Tango controller.

2. $MoSe_2$ was exfoliated onto silicon oxide and a suitable flake identified by optical microscopy. The crystal axes were determined by room temperature linear-polarization-resolved second harmonic generation (SHG) at normal incidence with reflection geometry (*26*–*28*) with excitation at 1.4 µm. The graphene-on-stamp was aligned to the $MoSe_2$ flake, pressed against it, heated to 90 °C and then peeled off to remove the $MoSe_2$ from the silicon oxide substrate, giving $MoSe_2$-on-graphene-on-stamp.

3. WSe_2 was exfoliated onto silicon oxide and a suitable flake identified by optical microscopy. Its crystal axes were also determined by SHG with excitation at 1.5 µm. The $MoSe_2$-on-graphene-on-stamp was aligned to the WSe_2 flake, the flake was rotated to align the $MoSe_2$ and WSe_2 armchair axes within 2 degrees, and dry transfer at 90 °C was used to form the WSe_2-on-$MoSe_2$-on-graphene stack on the stamp.

4. A thin flake of graphite was exfoliated onto a doped silicon substrate and identified by optical microscopy and atomic force microscopy. The stamp was aligned, and the stack pressed firmly against the graphite.

5. The substrate was heated to 150 °C and the PDMS stamp peeled off, leaving the stack and PC on the silicon. The sample was then cleaned, removing the PC, by a solvent wash in chloroform, rinsed in IPA, and dried under N_2 gas followed by thermal annealing in an Argon(95%)/Hydrogen(5%) atmosphere at 400 °C for 2 hr.

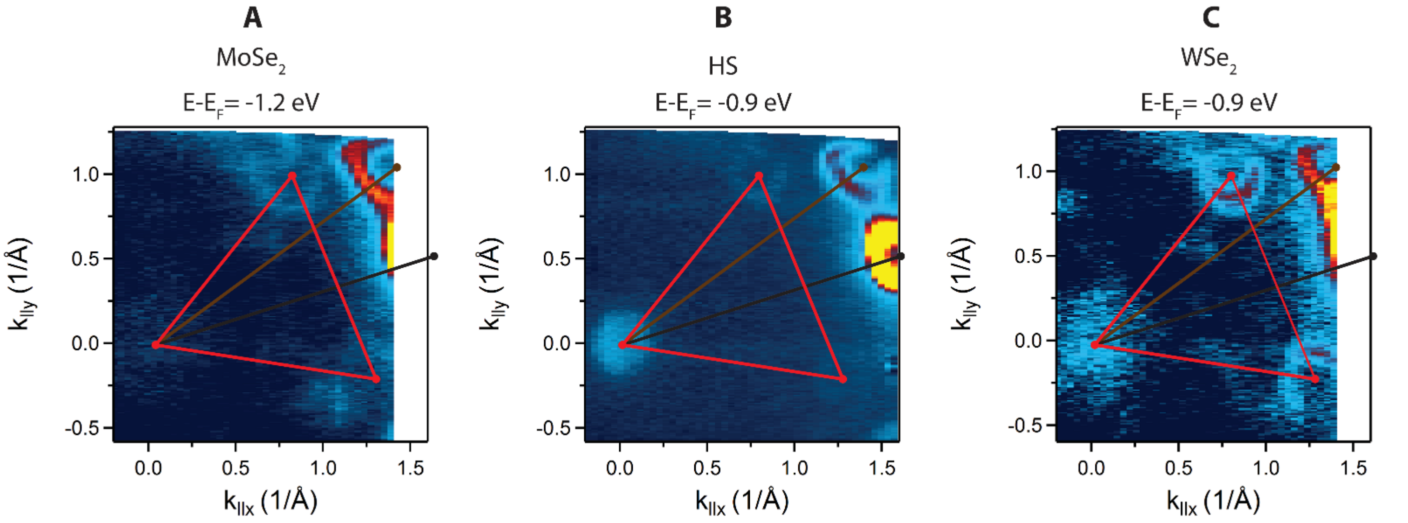

fig. S4. Relative orientations of the layers in an encapsulated $MoSe_2/WSe_2$ heterostructure. From Figure 2 of the main text. Constant energy slices with highlighted MX_2 Γ and K points (red circles connected by lines), graphene K point (black circle connected by line) and graphite K point (brown circle connected by line) from: (**A**) the $MoSe_2$ flake; (**B**) the heterostructure region; and (**C**) the WSe_2 flake. The MX_2 Γ and K points were accurately found by fitting line profiles and coincided to within a rotation of 1° and a relative reciprocal spacing of 1%.

section S3. Linear-scaling DFT calculations for twisted $MoSe_2/WSe_2$ heterobilayers

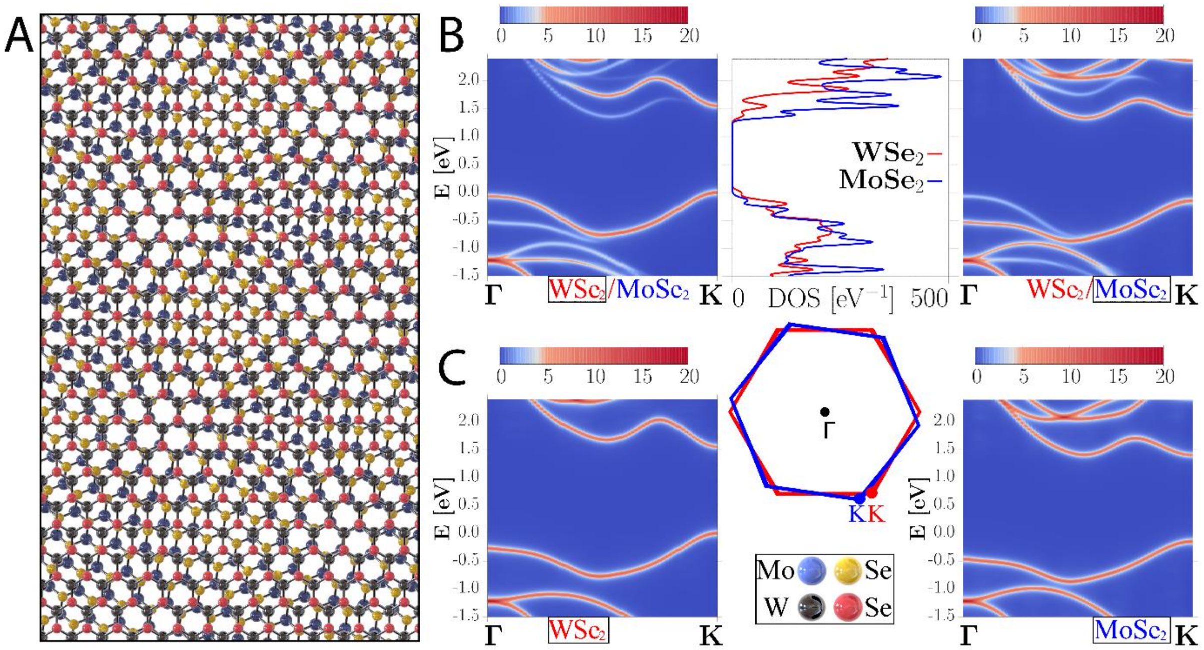

fig. S5. Linear-scaling DFT predictions of the band structure of the twisted $MoSe_2/WSe_2$ interface. (**A**) Atomic model of $MoSe_2/WSe_2$ heterostructure with a twist angle of 8.21°. (**B**) Unfolded spectral function of the twisted $MoSe_2/WSe_2$ heterostructure (A), projected on the WSe_2 monolayer (left) and $MoSe_2$ layer (right). The center inset shows the density of states (DoS) projected onto the WSe_2 and $MoSe_2$ layers from the heterointerface. (**C**) The spectral-function representation of the independent monolayers for WSe_2 (left) and $MoSe_2$ (right). The color scales for B and C are arbitrary, and reflect the effective DoS per supercell as projected onto the reduced Brillouin zone of the respective monolayer. The energy reference is the energy of the valence band maximum (VBM) at K in the heterostructure.

Misaligned (rotated) MX_2 layers form coincidence cells too large for the capabilities of plane-wave DFT. Instead, linear-scaling DFT was used to gain insight into the effects of hybridization on band edge energies for incommensurate structures. For full methodological details, see Supplementary section S7. We have previously shown that the energy landscape for twisted MX_2 heterostructures is roughly independent of twist angle, (*30*) and similarly the hybridization induced shifts at Γ are consistent in magnitude. For this reason, we considered the angle which resulted in the smallest supercell size; a simulation cell containing 873 atoms (432 for WSe_2, 441 for $MoSe_2$) for layers rotated by 8.21°, with strain < 1% (in the $MoSe_2$ layer). To observe the band structure effects of each layer in the presence of the other, we have calculated the unfolded spectral function, which was projected selectively on each of the component layers, as shown in fig. S5B and as described in detail in our previous works. (*30*)

Comparison between the band structures of the independent monolayers and the unfolded spectral-functions of the corresponding layers in the heterostructure shows low-spectral weight band intrusions from one monolayer into the other upon stacking. Moreover, the valence band maximum (VBM) at Γ of WSe_2 is raised by 202 meV, while the $MoSe_2$ VBM is lowered by 67 meV giving an increase in separation of ~ 250 meV. The experimental results show qualitatively similar behavior, with the bands shifting in the same direction and with a similar magnitude of increase in separation (experimentally ~ 100 meV) to the DFT predictions. Note that currently the linear scaling DFT approach adopted here does not include spin-orbit interactions – these are not expected to significantly alter the band structure at Γ but do change the band structure at K.

section S4. Band structure of twisted monolayer $MoSe_2/WSe_2$

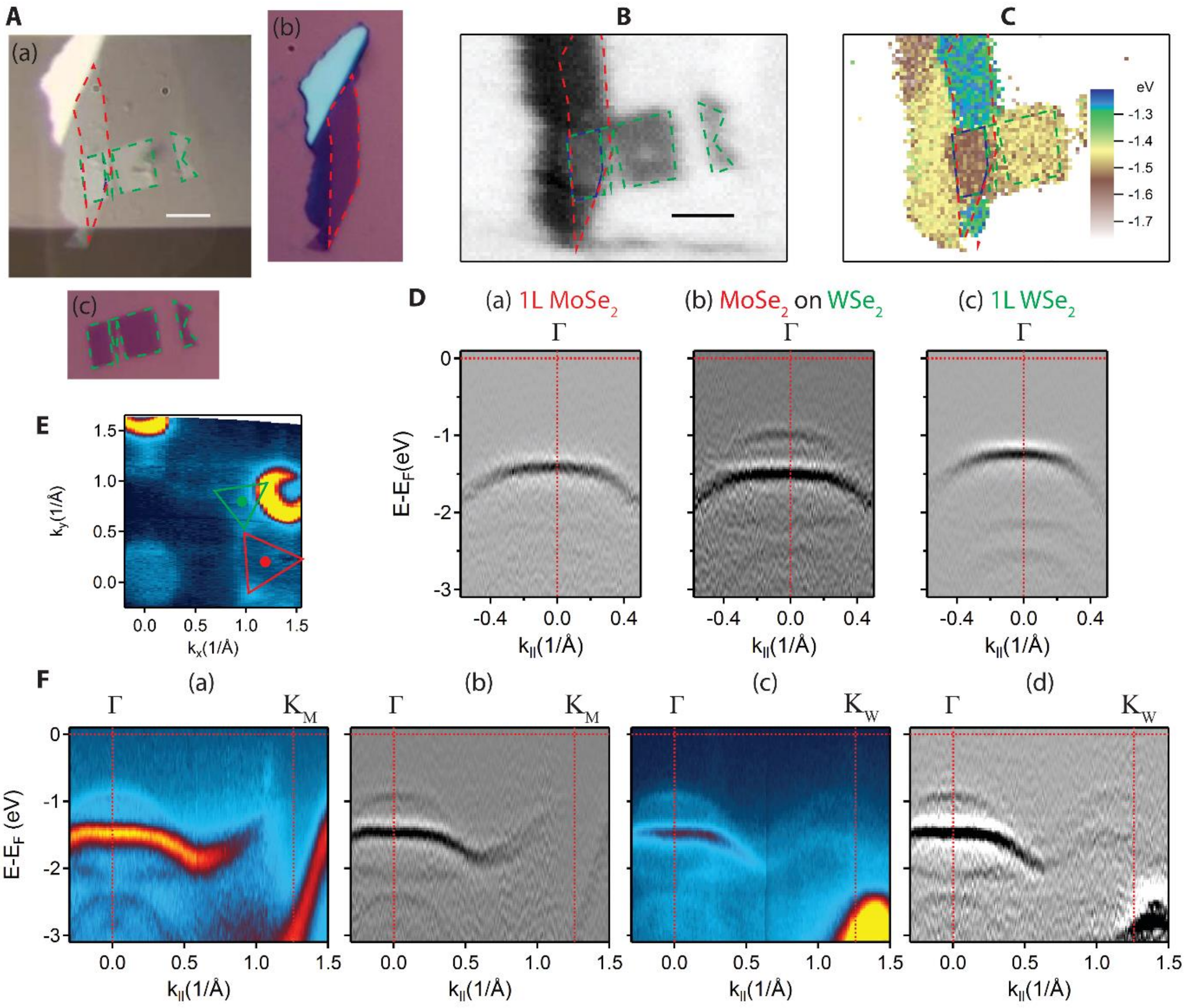

fig. S6. Band structure of a twisted monolayer $MoSe_2/WSe_2$ heterostructure. (**A**) Optical image of the heterostructure and its components; (**a**) the completed heterostructure consisting of graphene on $MoSe_2$ (green dashed line) on WSe_2 (red dashed line) on graphite on silicon, with (**b**) the WSe_2 flake and (**c**) the $MoSe_2$ flake on silicon oxide prior to transfer. The heterostructure region is outline in blue. (**B**) Integrated SPEM map at Γ near E_F; scale bar is 5 µm. (**C**) Corresponding map of the energy of maximum emission, showing the shift in energy due to hybridization in the heterostructure region. (**D**) Band dispersions around Γ from (**a**) the monolayer $MoSe_2$, (**b**) the heterostructure $MoSe_2$ on WSe_2, and (**c**) the monolayer WSe_2. The slices are in the k_y direction which here is not along a high symmetry axis. (**E**) Constant energy slice from full $\boldsymbol{E-k}$ spectra in the heterostructure region at $E\text{-}E_F = -0.78$ eV, $MoSe_2$ and WSe_2 K points are marked by green and red circles respectively. **F,** Slices from a full $\boldsymbol{E-k}$ spectra in the heterostructure region: (**a**) and (**c**) show intensity in the Γ to K direction for the $MoSe_2$ and WSe_2 respectively, (**b**) and (**d**) are the corresponding results differentiated twice with respect to energy.

A twisted bilayer of $MoSe_2$ on WSe_2, encapsulated between graphene and graphite, was also investigated. As shown in fig. S7, in the heterostructure region only two bands were seen at Γ, shifted from the bands in the independent monolayers: WSe_2 shifted up by 250 meV (cf 202 meV from the LS-DFT calculations in fig. S5) and $MoSe_2$ shifted down by 90 meV (cf 67 meV from LS-DFT). These shifts are significantly less than in the aligned heterostructure, where the WSe_2 was shifted up by >400 meV. The twist angle between the WSe_2 and $MoSe_2$ was measured to be 34° from the $\boldsymbol{E-k}$ spectra.

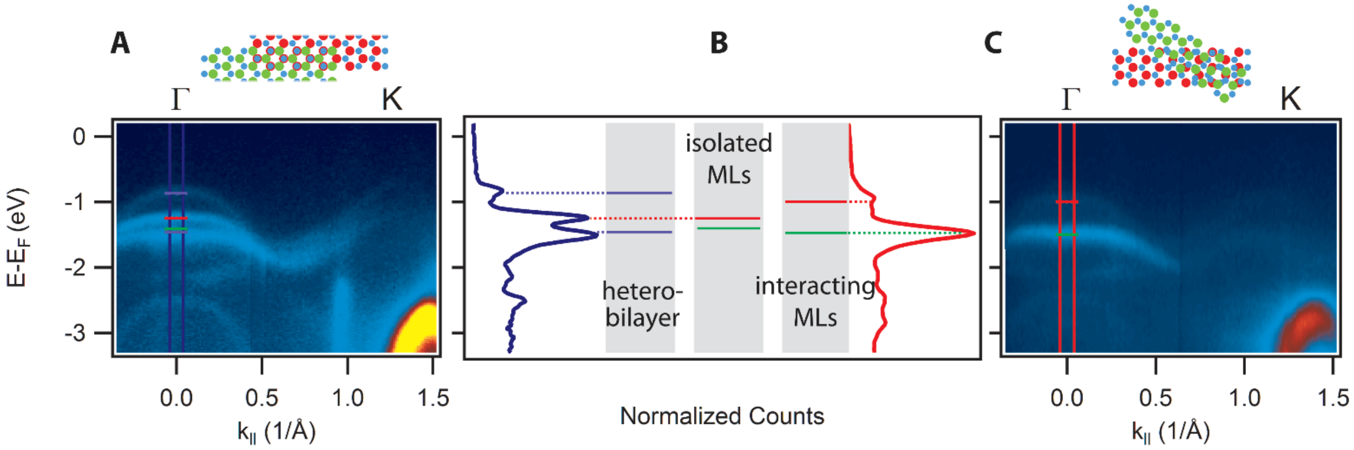

fig. S7. Comparison between bands and hybridization in aligned and twisted heterostructures. (**A**) raw ARPES data showing a momentum slice in the Γ-K direction for an aligned $MoSe_2$-on-WSe_2 heterostructure; an energy distribution curve (EDC) is extracted at Γ (from region marked by the two vertical blue lines) and is shown in (**B**). (**C**) raw ARPES data showing a momentum slice in the Γ-K (WSe_2) direction for a twisted $MoSe_2$-on-WSe_2 heterostructure; an EDC is also extracted at Γ (from region marked by the two vertical red lines) and is also shown in B. The DFT predicted band positions at Γ of the commensurate heterobilayer (two bands, marked in blue), and interacting monolayers (two bands, marked in red for WSe_2 and green for $MoSe_2$) are also shown in B, along with the band positions at Γ for the isolated monolayers monolayers (two bands, marked in red for WSe_2 and green for $MoSe_2$).

section S5. ARPES of encapsulated $MoSe_2/WSe_2$ with heterotrilayer regions

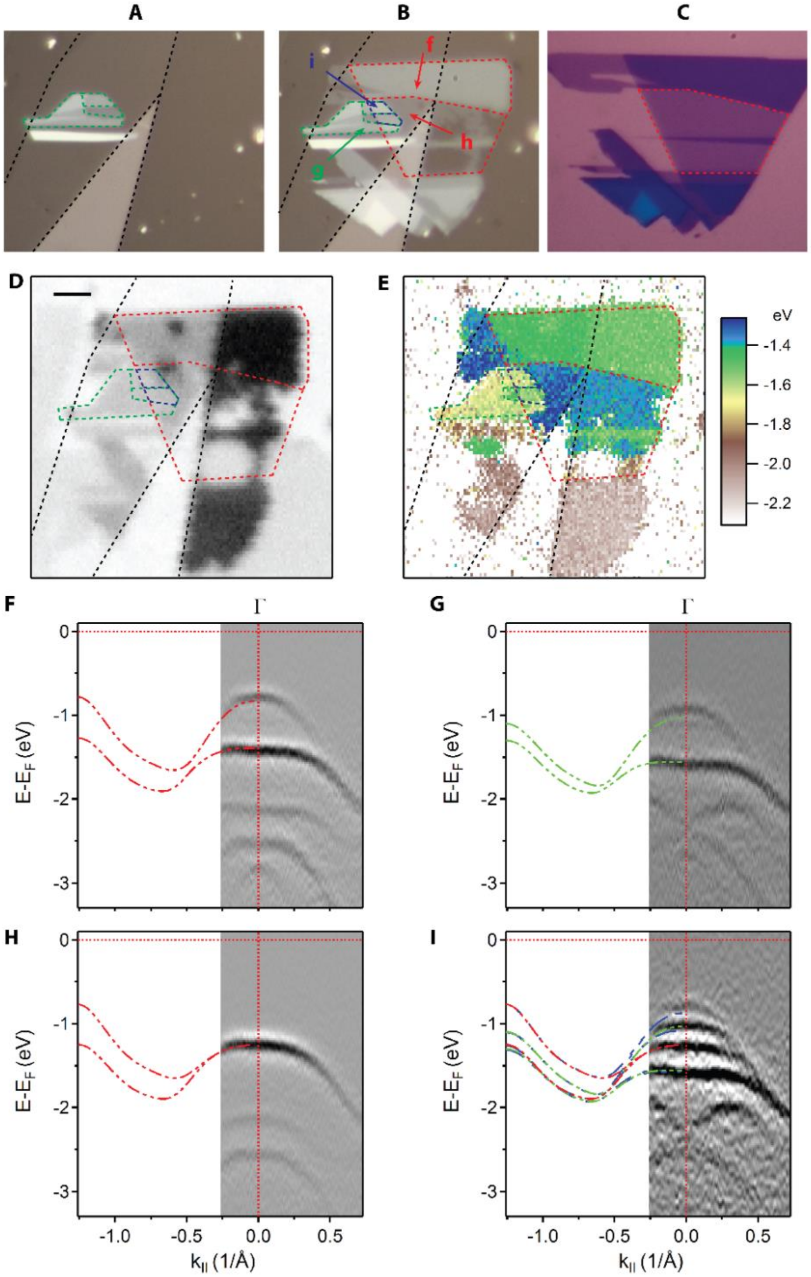

fig. S8. Bands and hybridization in a $MoSe_2/WSe_2$ structure with heterotrilayer regions. Optical images of: (**A**) the $MoSe_2$ flake (outlined by dashed green line) on graphene (outlined by dashed black line) on the stamp prior to transfer with the heterostructure region outlined in blue; (**B**) the WSe_2 (outlined by dashed red line), $MoSe_2$ and graphene on the stamp, and (**C**) the exfoliated WSe_2 flake on silicon oxide prior to transfer. (**D**) Integrated SPEM map at Γ near E_F; scale bar is 5 μm. (**E**) Corresponding map of the energy of maximum emission. The dispersion around Γ is shown in (**F** to **I**) from points in the bilayer WSe_2, bilayer $MoSe_2$, monolayer WSe_2, and the heterotrilayer (bilayer $MoSe_2$ on monolayer WSe_2) regions respectively (labeled in B) with corresponding DFT calculations overlaid: for the heterotrilayer the independent layers (monolayer WSe_2 red dashed, bilayer $MoSe_2$ green dashed) and commensurate heterotrilayer (blue dashed) are both shown. Unfortunately for this sample the drift in position during acquisition was too quick to acquire full $\boldsymbol{E}-\boldsymbol{k}$ spectra in each region, so these dispersions are not from high symmetry directions.

section S6. Exciton energies at lower temperatures

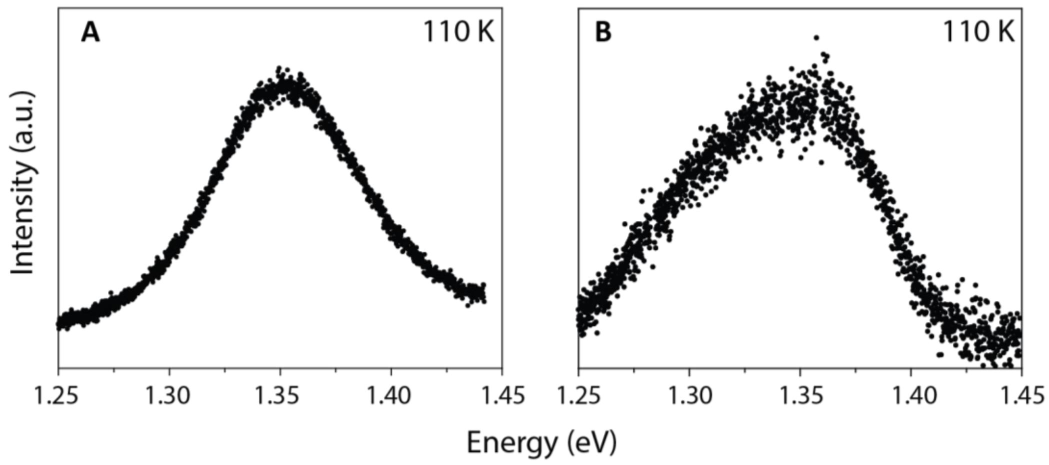

fig. S9. Lower-temperature interlayer exciton photoluminescence. (**A** and **B**) Interlayer exciton photoluminescence spectra from two different samples at 110 K.

ARPES experiments were performed at 110 K, while the PL measurements were taken at room temperature (295 K). Here we analyze how this affects our determination of the interlayer exciton binding energy. The intralayer PL peaks $\hbar\omega(\mathrm{X_M})$ and $\hbar\omega(\mathrm{X_W})$ blueshift significantly as the temperature decreases. In particular, $\hbar\omega(\mathrm{X_M})$ is known to increase ~60 meV from 295 K to 110 K and an additional 10 meV from 110 to 30 K (*49*). However, the interlayer peak $\hbar\omega(\mathrm{X_I})$ does not shift as much with temperature. In 10 of our 13 samples from Fig. 4, the average $\hbar\omega(\mathrm{X_I})$ at low temperature (5-30 K) is ~1.36 eV, a 20 meV blue shift from 295 K. Thus the energy separation between $MoSe_2$ intralayer PL and $\mathrm{X_I}$ tends to increase at lower temperature, which decreases our estimate of δE_b. Consequently, our main result of a lower limit on the interlayer binding energy $E_b(\mathrm{X_I}) = E_b(\mathrm{X_M}) - \delta E_b \geq 300\ \mathrm{meV} - (\Delta_{\mathrm{VBO}} - [\hbar\omega(\mathrm{X_M}) - \hbar\omega(\mathrm{X_I})])$ is in fact a relatively conservative estimate.

In addition, we measured two samples at ~110 K and found $\hbar\omega(\mathrm{X_I})$ near 1.35 eV (fig. S10), which suggests $\hbar\omega(\mathrm{X_M}) - \hbar\omega(\mathrm{X_I}) \approx 290$ meV (*50*) and thus $E_b(\mathrm{X_I}) \geq 290$ meV. Due to the uncertainty in $\hbar\omega(\mathrm{X_I})$, we retain our estimate that $E_b(\mathrm{X_I}) \geq 200$ meV, the main point being that the interlayer and intralayer binding are on the same order.

section S7. DFT methodology

Plane-wave DFT: for calculations involving individual materials and aligned heterostructures, the Quantum Espresso (*42*) plane-wave DFT package was used. The ultrasoft atomic datasets of Garrity et al (*45*) were used for structural calculations, and the optB88-vdW functional (*46*) was employed, due to its previous success in describing interlayer interactions in 2D materials. (*30*) The structures were optimized until forces were smaller than 10^{-4} Ry / Bohr for monolayers, and 5×10^{-4} Ry / Bohr for bilayers and bulk, while stresses were required to be smaller than 0.05 GPa. Subsequently, the band structures were calculated using the high-accuracy fully-relativistic PAW potentials of Dal Corso, (*43*) such that spin-orbit interaction was included. We used a 12x12 in-plane k-point sampling grid (with 4 out-of-plane k-points for the bulk), an 800 eV plane-wave energy cutoff, and an 8000 eV charge density cutoff. The simulation cell height was 30.0 Å, to avoid interaction between periodic images. All these parameters were determined to be sufficient for very good convergence of structural and electronic properties.

Linear-scaling DFT: we utilized the ONETEP code, (*31*) which uses an efficiently-parallelized linear-scaling formalism (*47*) based around representation of the single-electron density matrix via in-situ optimized local orbitals and sparse matrices. Once again we used the optB88-vdW functional and a kinetic-energy cutoff of 800 eV. ONETEP does not currently have the ability to include spin-orbit coupling, the projector-augmented wave (PAW) method was employed, with atomic datasets exactly equivalent to the ultra-soft pseudopotential (USPP) datasets used for the geometry optimizations in the plane-wave DFT calculations described above. The Mo and W atoms both contained 14 valence electrons ($4s^2$,$4p^6$,$4d^5$,$5s^1$ for Mo, $5s^2$,$5p^6$,$5d^4$,$6s^2$ for W), while S and Se contained only 6 valence electrons ($3s^2$,$3p^4$ for S, $4s^2$,$4p^4$ for Se).

ONETEP uses a nested-loop optimization scheme in which an outer loop optimizes the form of the local orbitals, while an inner loop optimizes the density matrix for fixed local orbitals. The flexibility provided by in-situ optimization means that it is possible to use relatively small number of local orbitals and retain systematically controllable accuracy equivalent to the plane-wave approach. In this case we used 13 non-orthogonal Wannier functions (NGWFs) for W and Mo (10 for the valence electrons, 3 allowing for additional polarization) and 9 for S and Se (4 for the valence electrons, 5 for additional variational freedom). All NGWFs were chosen to have a large cut-off radius (13.0 bohr), and the convergence criterion was that the root mean square of the NGWF gradient be smaller than 2×10^{-6}. For each NGWF optimization step, 8 self-consistent density-kernel iterations were performed. Truncation of the density kernel was not necessary for the system sizes employed. Geometry optimization was performed by relaxing the internal atomic coordinates until the forces (*48*) were below 0.1 eV / Å. The supercell was constructed by first determining the coincidence cells of the over-lapping rotated monolayers, allowing a maximum of 1% strain. Spectral functions were calculated by unfolding supercell eigenstates into the primitive cells of each layer, as described in previous work. (*30*)
